# Diagnostic value of chest ultrasound after cardiac surgery: a comparison with chest X-ray and auscultation

**DOI:** 10.1186/cc13320

**Published:** 2014-03-17

**Authors:** A Vezzani, T Manca, F Benassi, A Gallingani, I Spaggiari, C Brusasco, F Corradi, T Gherli

**Affiliations:** 1 Azienda Ospedaliero Universitaria di Parma, Italy; 2 Università degli studi di Genova, Italy; 3 E.O. Ospedali Galiera, Genova, Italy

## Introduction

Chest auscultation and chest X-ray are commonly used to detect postoperative abnormalities and complications in patients admitted to intensive care after cardiac surgery [1],[2]. The aim of the study was to evaluate whether chest ultrasound represents an effective alternative to bedside chest X-ray to identify early postoperative abnormalities.

## Methods

A total of 151 consecutive patients (103 male and 47 female) were studied by chest auscultation, ultrasound and X-ray upon admission to intensive care after cardiac surgery. Six pathologic entities were explored by each method: postero-lateral pleural effusion and/ or alveolar consolidation (PLAPS), alveolar-interstitial syndrome (AIS), alveolar consolidation (AC), pneumothorax (PTX), pleural effusion (PE), and pericardial effusion with or without cardiac tamponade. Positions of the endotracheal tube and central venous catheter were also checked.

## Results

Ninety-four of the 151 patients included (62%) showed abnormalities on chest X-ray (AC 9%, AIS 25%, PLAPS 42%, PE 3.3%, PTX 2%). Compared with chest X-ray, chest ultrasound had a sensitivity of 86% and a specificity of 99% for AC, a sensitivity of 95% and a specificity of 100% for AIS, a sensitivity of 97% and a specificity of 98% for PLAPS, a sensitivity of 99% and a specificity of 100% for PE, and a sensitivity and specificity of 100% for PTX. Furthermore, chest ultrasound detected all pericardial effusions while neither chest X-ray nor chest auscultation were able to identify them. Chest ultrasound identified all cases of endotracheal tube (two patients) and central venous catheter (two patients). There was a highly significant correlation between abnormalities detected by chest ultrasound and X-ray (*k *= 0.90), but a poor correlation between chest auscultation and X-ray abnormalities (*k *= 0.15).

## Conclusion

Chest auscultation may help identify endotracheal misplacement and tension pneumothorax but it may miss most of major abnormalities. Chest ultrasound represents a valid alternative to chest X-ray to detect all postoperative abnormalities and misplacements.
